# Digital health literacy—a key factor in realizing the value of digital transformation in healthcare

**DOI:** 10.3389/fdgth.2025.1461342

**Published:** 2025-06-05

**Authors:** Sarah Wamala Andersson, Marta Pisano Gonzalez

**Affiliations:** ^1^ School of Health, Care and Welfare, Mälardalen University, Västerås, Sweden; ^2^ Department of Care and Social Healthcare, Ministry of Health, Oviedo, Spain

**Keywords:** digital health, equity, digital transformation (DT), digital health literacy, health care

## Abstract

**Background:**

Digital health technologies and AI are transforming healthcare by improving access, optimizing care, and enabling personalized, preventive, and predictive solutions. However, digital health literacy remains a critical barrier, affecting individuals' ability to engage with digital health technologies (DHTs) and limiting progress toward digital health equity.

**Aims:**

To propose a framework that captures the complexity of digital health literacy and guides research, and to share key insights from the Improving Digital Empowerment for Active Healthy Living EU project.

**Results:**

We introduce a conceptual framework that explores digital health literacy's interactions with social determinants, providing a foundation for research, policy, and practice. Insights from the project (Improving Digital Empowerment for Active Healthy Living), involving 14 partners across 10 European countries, offer evidence-based strategies to empower individuals and promote digital inclusion.

**Concluding remarks:**

To keep pace with technological advancements, digital health literacy should be integrated into lifelong learning initiatives. Urgent research is needed to inform policies and guide interventions that enhance digital health literacy and ensure equitable digital transformation in healthcare.

## 1 Introduction

As global life expectancy continues to rise, maintaining good health has become an increasingly sought-after goal for many, with technological advancements offering promising solutions. Digital Health Technologies (DHTs) have the potential to revolutionize healthcare by enabling virtual care, remote monitoring, improved disease management, and more personalized treatment options (1, 2).

DHTs encompass telemedicine, mobile applications, wearable devices, electronic health records, and artificial intelligence (AI) (3, 4). These technologies facilitate real-time data collection on patients' vital signs, lifestyles, and medical histories, supporting more effective care pathways, personalized treatments, and predictive healthcare. Moreover, DHTs and AI have the potential to reduce healthcare costs while simultaneously improving the quality of care (5).

However, one significant barrier to fully leveraging the potential of DHTs is digital health literacy. Without sufficient understanding of how to use these technologies, their value in transforming healthcare cannot be fully realized, hindering efforts toward achieving digital health equity. Additional challenges include insufficient research-based evaluations, skepticism, resistance to change, and limited access to digital tools (6, 7). Digital health literacy is a multifaceted concept, requiring a comprehensive approach to ensure better outcomes (8).

The World Health Organization (WHO) (6) has underscored the importance of developing and implementing digital health technologies in ways that promote equity, affordability, and accessibility. In its Regional Digital Health Action Plan 2023–2030, the WHO highlights Digital Health Literacy (dHL) as a critical factor in achieving universal health coverage and ensuring that all populations benefit from digital health solutions (7).

The WHO defines dHL as the ability to seek, find, understand, and appraise health information from electronic sources, and to apply that knowledge to solve health-related problems (7). Achieving dHL is therefore essential for both patients and healthcare professionals, enabling them to effectively access, evaluate, and apply health information from digital platforms. This requires not only technical skills for operating digital tools but also cognitive skills for interpreting and communicating health data (9). Consequently, digital health literacy should be incorporated into lifelong learning initiatives (7, 10).

Policymakers, healthcare professionals, and technology developers are increasingly acknowledging that the successful integration of technology and innovation into healthcare goes beyond merely financial investment. It also necessitates a concentrated effort on enhancing the usability and accessibility of these solutions, ensuring that they are not only effective but also user-friendly and equitable for all populations (11).

The future of healthcare delivery is expected to shift towards empowering patients to take control of their health management using DHTs, facilitating better communication with healthcare providers and professionals.

This paper highlights the crucial role of Digital Health Literacy (dHL) in unlocking the full potential of Digital Health Technologies (DHTs) such as telemedicine, mobile apps, wearables, and AI to transform healthcare. Despite their promise, barriers like low dHL, inadequate evaluations, and limited access impede widespread adoption.

The paper examines the connection between dHL and health equity, proposing a framework to address the digital health equity gap, demonstrates insights from the IDEAHL project, a European initiative dedicated to advancing digital empowerment for healthy living and discusses the way forward.

The structure is as follows: the paper first provides an overview of DHTs and their challenges, followed by a review of research gaps and policy needs, and a conceptual framework for addressing dHL. It then presents key findings from the IDEAHL project and concludes with actionable strategies to accelerate the digital transformation of healthcare.

### 1.1 Gaps and needs

Despite the increasing recognition of digital health literacy (dHL) and its significance, there remains a notable deficiency in effective policies and interventions aimed at enhancing dHL among both healthcare professionals and patients. A 2023 WHO study (7) highlighted the need for improved training programs, incentives, and evaluations to boost the adoption of digital health tools among healthcare workers, emphasizing existing barriers in this area.

Furthermore, a recent systematic review (10) assessed the effectiveness of digital health interventions in improving health literacy and found that while such interventions have potential, their impact varies due to factors such as the digital divide, age, and socioeconomic status, indicating a lack of universally effective strategies. In fact, only about half of the countries in Europe and Central Asia have developed policies for digital health literacy and implemented digital inclusion plans, leaving millions without adequate support (7).

Thus, there is a critical need for developing and implementing effective policies and interventions to enhance digital health literacy among both healthcare professionals and patients.

Digital health literacy is complex (10) as it includes other types of literacy and other components related to social determinants of health and social context (12–14) Thus, addressing dHL requires coordination across three critical levels: individual (micro), institutional (meso), and policymaking (macro) levels. To date there is a lack of comprehensive research-based tools to measure dHL at individual, organizational and policy levels (15–18).

Vulnerable people include those with low socioeconomic status, elderly persons and persons living with disabilities, chronic diseases and mental health conditions are more likely to experience digital exclusion (19, 20) as they also tend to have higher disease burdens and health needs yet often lack access to or engagement with DHTs. Low levels of dHL limit the delivery of person-centered care and the opportunity for patients to make informed decisions (4, 5). This ultimately creates digital exclusion and digital health equity gap.

Thus, digital health literacy (dHL) and health equity are intrinsically linked, collectively shaping health disparities. This interconnection positions dHL as a “super determinant” of health, influencing individuals' ability to access, understand, and utilize digital health resources effectively” (12, 21, 22). Moreover, dHL plays a pivotal role in ensuring the equitable distribution of health services in the era of digital transformation by mitigating barriers to digital access and fostering inclusive health communication strategies (18).

Thus, dHL and equity are closely linked and together they contribute to health disparities, which makes dHL a super determinant of health (12, 18, 21, 22) and playing a key role in ensuring equitable distribution of health services in the era of digital transformation (23).

According to the WHO (7), only half of the countries in Europe and Central Asia have implemented policies to enhance digital health literacy (dHL), leaving millions at risk of exclusion from digital health advancements. As technology increasingly facilitates data collection, care coordination, and telemedicine, the lack of equitable digital access threatens to exacerbate health disparities.

To ensure that digital health does not become a driver of inequality, policies that actively promote dHL must be prioritized (2). However, there remains a significant gap in the development and implementation of effective strategies to address digital health inequities.

The IDEAHL project was launched to address existing gaps in digital health literacy (dHL) measurement, interventions, and policy development. Funded by Horizon Europe (GA 101057477) (24), the project's primary aim was to empower EU citizens to take an active role in managing their health and well-being through digital tools, while also promoting social innovations that enhance person-centered care models. To develop a comprehensive EU dHL Strategy, a collaborative co-creation process was initiated, involving 140 sessions with 1,434 participants from 19 distinct target groups. These included citizens, patients, healthcare and social service professionals, policymakers, non-health sector experts, and academics. Twelve pilot actions were carried out across ten participating countries, generating valuable data that will serve as the basis for developing a framework and indicators to assess digital health literacy.

## 2 Observations

### 2.1 Understanding the complexity of dHL using a conceptual framework

Advancing digital health equity and accelerating digital transformation in healthcare requires a nuanced approach to understanding digital health literacy (dHL), especially as it evolves in response to technological advances and shifting societal contexts (20, 22, 25–27). dHL is not only about technical skills but is intricately linked to the social determinants of health, such as education, socioeconomic status, and access to technology (10, 20, 28). As technology rapidly progresses and societal adoption varies, the concept of dHL is continuously shaped, highlighting the need for a deeper understanding of how digital literacy interacts with health equity (28).

**Figure 1:**
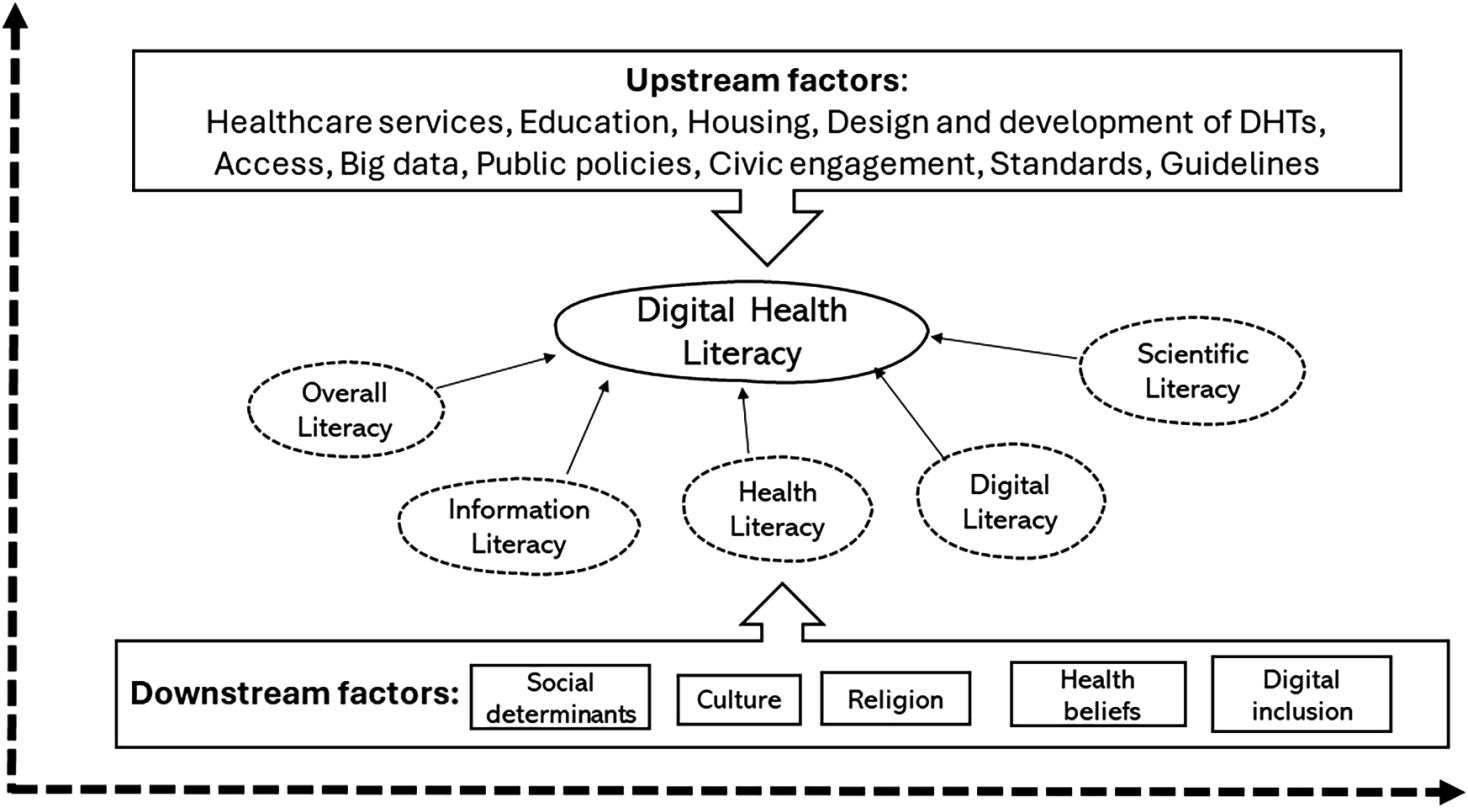
Complexity of dHL as a concept and levels of intervention.

This conceptual framework (Figure 1) aims to guide the development of policies and interventions to enhance dHL across diverse populations. By addressing the multifaceted barriers related to dHL, such as access to technology, health education, and infrastructure, policymakers can help bridge the digital divide, promoting equitable access to digital health resources and ensuring improved health outcomes in an increasingly digitized healthcare environment (29).

To offer a structured understanding of dHL and the barriers that need to be tackled, we propose a conceptual framework (Figure 1), which extends the Lily model of eHealth literacy (10). This model incorporates five core skill sets essential for digital health literacy: overall literacy, information literacy—Skills to access, evaluate, and use information effectively, health literacy—Understanding and processing health-related information, digital literacy and scientific literacy (Table 1). Thus, this framework (Figure 1) illustrates the dynamic interplay between social determinants of health (SDH) and health literacy at the individual level (30), underscoring their reciprocal influence (31).

**Table 1:** Components of digital health literacy.

Components	Definition
Overall literacy	Basic literacy skills, also referred to as civic literacy
information literacy	Ability to search, filter, analyze, understand and transfer information to real-life implementation and practice
Health literacy	Understanding basic health information and making health-related decisions
Digital literacy	Access, confident and critical use of a digital technologies for information, communication and basic problem-solving in all aspects of life
Scientific literacy	Capacity to identify questions, understand and create knowledge in a systematic manner and make conclusions on real-life actions

Together, these competencies shape an individual's capacity to navigate, interpret, and apply digital health resources effectively, ultimately promoting greater digital health equity (28).

The relationship between dHL, digital exclusion, and health equity is particularly important but requires further exploration. The operational aspects of dHL, such as the technical and cognitive skills involved, must be addressed in greater detail to clarify how they interact and influence health outcomes. For example, individuals with higher digital literacy may be better equipped to access health information, whereas those lacking such skills may face digital exclusion, exacerbating health disparities (10, 22, 30, 32).

The framework depicted in Figure 1 illustrates the dynamic interaction between social determinants of health (SDH) and health literacy at the individual level (30), emphasizing their bidirectional influence (32). It also underscores the importance of addressing both upstream structural factors (such as policies, socioeconomic conditions, and digital infrastructure) and downstream individual-level factors (such as digital skills, technology access, and personal health behaviors) in order to drive meaningful and sustainable improvements in digital health literacy (dHL) (32). By adopting this holistic approach, the framework seeks to advance health equity and ensure that digital health solutions are accessible and advantageous to all (6, 33) and support future research initiatives.

### 2.2 Key lessons from the EU-IDEAHL project—improving digital empowerment for active healthy living

The IDEAHL (Improving Digital Empowerment for Active Healthy Living) is a newly finalized project financed by Horizon Europe with 14 partners from 10 European countries. The aim was to develop and test new models and approaches of (digital) health literacy dHLdHL intervention through the co-creation of a comprehensive and inclusive EU dHL Strategy (34, 35).

*Key lessons from the IDEAHL project regarding addressing dHL are summarized below.*

#### 2.2.1 Establishing cross-border multi- and interdisciplinary sciences

The digital health landscape is inherently complex and requires the combined expertise of various disciplines (36, 37). This requires a broad consideration of medical, technical, contextual, social and cultural aspects, including adapting language and communication, involving the community, analyzing socio-economic and cultural aspects, focusing on prevention and health promotion, and using accessible technology.

Additionally, ensuring support from the EU, national, and local policy makers and engaging both health and non-health sectors along with citizens is crucial. Creating platforms for cross-border and interdisciplinary partnerships are needed to share best practices, develop collaborative approaches and drive future innovations, while ensuring scaling up of current technologies.

The multi- and interdisciplinary scientific approach within the IDEAHL consortium, including ten countries Belgium, Denmark, Finland, France, Germany, Ireland, Italy, Portugal, Spain and Sweden was a success factor in conducting several studies related to evidence generation, co-creation workshops, dissemination, piloting, implementation and evaluation (34, 35).

#### 2.2.2 Measuring dHL levels at population level across countries

Measuring dHL at population level provides decision-makers with data-driven insights in shaping effective policies and interventions that enhance dHL across populations (8, 13, 16, 37–39).

The IDEAHL scoping review highlights a critical gap: the lack of a comprehensive, standardized tool to assess dHL across the EU. In the absence of a unified instrument, it is difficult to gather comparable data across different countries and regions (40). While IDEAHL identified seven existing tools designed to measure dHL in various target populations, none adequately addressed the need for population-level assessments across the EU.

In a recent systematic review conducted as part of the IDEAHL project (41), we found that the eHealth Literacy Scale (eHEALS), though widely used, has limitations in its scope and adaptability. Future tools should be developed to better reflect emerging digital trends, considering factors such as individual skills, context-specific elements, health systems, the specific technologies in use, and user interfaces. The primary objective should be improving health outcomes, rather than solely increasing dHL levels. Moreover, the development of digital health literacy tools must integrate ethical considerations, including privacy and data security.

The WHO report (6) outlines strategies for incorporating digital health into healthcare systems globally and underscores the need to measure and understand dHL to develop sustainable digital health policies and interventions. Evaluating the impact of these policies and interventions on dHL trends allows for tracking progress over time and enables targeted support and resource allocation to ensure that interventions reach those most in need.

EU member states should collaborate with organizations such as Eurostat to conduct regular, standardized assessments of digital health literacy (dHL). This collaboration would facilitate the sharing of best practices and resources, promote a coordinated approach to improving dHL across the region, and generate real-world evidence to inform policy development (38–40).

#### 2.2.3 Understanding the needs of vulnerable population through co-creation methodologies

Effectively addressing digital health literacy (dHL) challenges within vulnerable populations requires a comprehensive understanding of their unique needs, technology access, and the barriers they encounter (22, 42, 43).

In the context of the IDEAHL project, co-creation methodologies were employed to directly engage vulnerable populations in the design and development of dHL interventions. This process facilitated an in-depth, empathetic understanding of their lived experiences and perspectives, ensuring that interventions were not only relevant but also accessible, effective, and respectful of the social, cultural, and environmental contexts in which these populations live. By engaging these communities directly, the project fostered solutions that were co-designed, empowering individuals to take ownership of their health and well-being (24).

The IDEAHL co-creation workshops were carefully tailored to be culturally and linguistically sensitive, creating a space where participants could openly share their cultural practices, trust concerns, and health-related beliefs surrounding technology. These activities spanned 10 countries and included 14 project partners, resulting in 140 co-creation sessions with 1,434 participants from 19 distinct target groups.

Rooted in the principles of participatory design and action research, the co-creation methodology emphasizes the importance of involving stakeholders at every phase of the project. Widely recognized as a powerful tool for fostering innovation, enhancing the effectiveness of interventions, and ensuring equitable health and social solutions, co-creation has proven to be an essential strategy in addressing the unique needs of vulnerable populations (44, 45).

In our recent study, conducted as part of the IDEAHL project (35), participants highlighted the critical need to offer targeted support to vulnerable groups, particularly those at risk of digital exclusion, to improve their digital health literacy.

#### 2.2.4 Implementing a comprehensive EU dHL strategy

The EU digital health literacy (dHL) strategy was developed through co-creation sessions with diverse stakeholders, ensuring it reflects real-world needs and experiences (46). It takes a multi-level approach, targeting dHL at the micro (individual), meso (organizational), and macro (policy) levels, with actionable recommendations primarily at the meso and macro levels. At the macro level, this includes integrating dHL into public health policies and fostering cross-sector collaborations. At the meso level, it focuses on incorporating dHL training into professional development and creating systems to help citizens navigate digital health resources. Successful implementation will require collaboration across all levels, infrastructure development, and fostering a culture of lifelong learning in dHL.

#### 2.2.5 Monitoring and evaluating dHL impact

Implementing the EU digital health literacy (dHL) strategy is essential for improving health outcomes across Europe, but it requires effective monitoring and evaluation mechanisms (46). A comprehensive framework has been developed, with guidelines, indicators, and tools to assess the impact of dHL interventions, accounting for geographic and socioeconomic factors that contribute to digital health inequalities. To aid assessment, the Global Atlas of Literacies for Health (GALH) (47) has been introduced, offering cross-country comparisons and serving as a guide for policymakers and health practitioners in designing and implementing effective dHL policies.

## 3 Discussion—ways forward with digital health literacy

### 3.1 Need for a holistic framework for bridging the digital health equity gap

The framework proposed in this paper highlights the dynamic relationship between social determinants of health (SDH) and individual-level health literacy, emphasizing their bidirectional influence (31). It stresses the need to address both upstream structural factors and downstream individual-level factors to foster meaningful and sustainable improvements in digital health literacy (dHL). By integrating these interconnected elements, this framework offers a comprehensive approach to guide policy development and practical interventions. Furthermore, it serves as a foundation for future research aiming at narrowing the digital health equity gap.

### 3.2 Adapt to key lessons from the IDEAHL project

To effectively enhance dHL across Europe, integrating key insights from the IDEAHL project (24, 34, 41) can be a good start. This includes fostering cross-border, multi- and interdisciplinary collaborations, systematically measuring dHL levels across the EU, addressing the specific needs of vulnerable populations, implementing the proposed EU dHL Strategy, and continuously monitoring and evaluating the impact of interventions and policies. By adopting these lessons, stakeholders can create a more inclusive, evidence-based, and sustainable approach to improving dHL and empowering individuals to navigate the digital health landscape effectively.

### 3.3 Fit-for-purpose training for healthcare professionals to support patient-centered digital health care

Enhancing dHL among healthcare professionals is essential for delivering truly patient-centered care in an increasingly digitalized healthcare landscape (6, 48).

As frontline providers, they play a crucial role in bridging the digital divide and supporting patients in navigating digital health tools effectively. However, many lack the necessary training to meet these evolving demands, highlighting the urgent need for “fit-for-purpose” dHL education (49). Integrating digital health competencies into healthcare curricula ensures professionals are equipped to enhance patient engagement, improve health outcomes, and address equity challenges (13). A comprehensive training approach should include proficiency in digital tools, patient education strategies, equity-focused interventions, and continuous learning to keep pace with technological advancements (50). By strengthening healthcare professionals' digital competencies, we can create a more inclusive, accessible, and patient-centered healthcare system.

### 3.4 Address the digital health equity gap through stakeholder engagement

Healthcare systems often operate in silos with fragmented strategies, failing to address long-term societal needs (51). As digital transformation accelerates, inclusive stakeholder collaboration is essential to closing the digital health equity gap. The Quadruple Helix Model, integrating the public sector (policy and funding), industry (technological innovation), academia (scientific research), and civil society (patient advocacy), provides a structured, holistic approach to developing sustainable, evidence-based digital health solutions that address diverse population needs (52, 53).

## 4 Concluding remarks

Digital transformation in healthcare offers significant opportunities to expand access, especially for vulnerable populations facing barriers to traditional care (22, 54). However, its success depends on the effective implementation and adoption of technologies, with digital health literacy (dHL) playing a key role (6, 7, 11).

The conceptual framework in this paper aims to guide digital health equity strategies, with the IDEAHL EU Digital Health Strategy serving as a model for policy development (49). As technology advances rapidly, integrating dHL into lifelong learning is crucial for ongoing adaptation and inclusion.

Urgent research is needed to inform policies that strengthen dHL at the national level, ensuring equitable benefits from digital transformation (55). Policymakers must prioritize digital health literacy, focusing on tailored information, digital support for prevention, and ethical considerations (6).

## Data Availability

Publicly available datasets were analyzed in this study. This data can be found here: NA.
